# Supplementary figures and images for: PAK1 regulates oligodendroglial proliferation and repopulation in homeostatic and demyelinating brain
Source: Cell Mol Life Sci. 2025 Jun 28;82(1):260. doi: 10.1007/s00018-025-05728-3 (PMC12209112; doi:10.1007/s00018-025-05728-3)

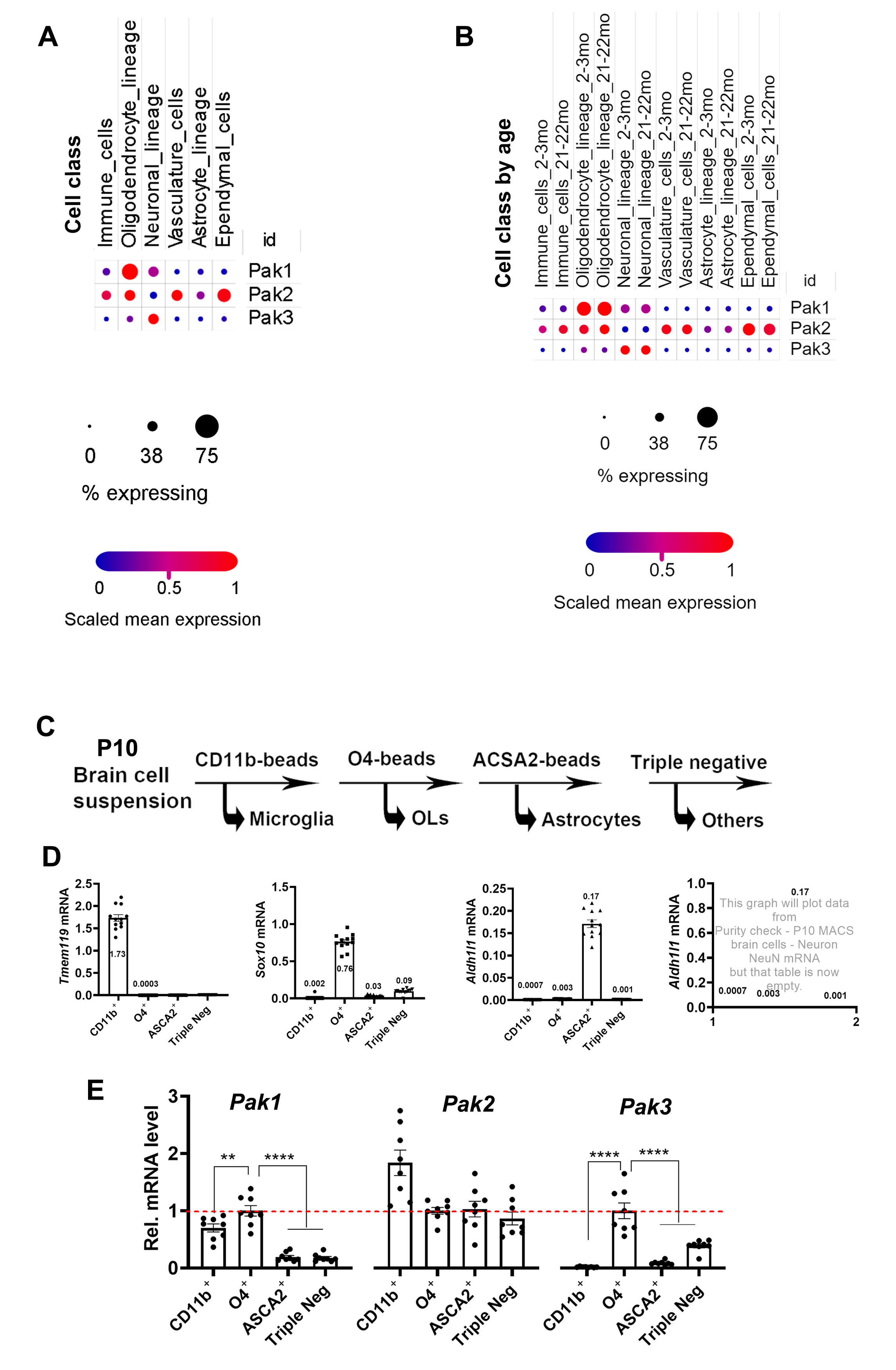

Supplement: Supplementary file 6 — Supplementary file5 (TIF 23530 KB) [file 18_2025_5728_MOESM5_ESM.tif]

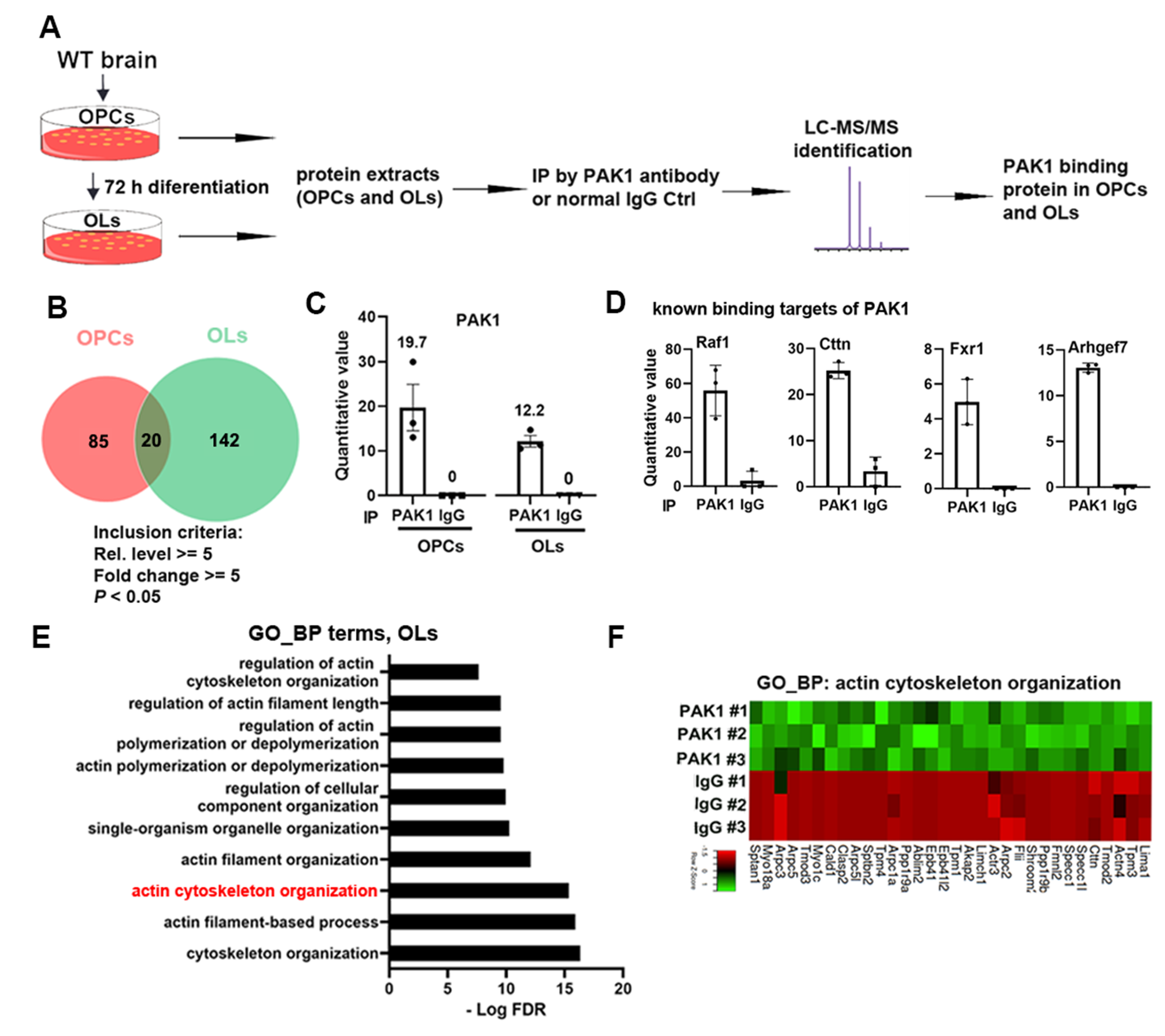

Supplement: Supplementary file 7 — Supplementary figure 2 [file 18_2025_5728_Fig6_ESM.png]

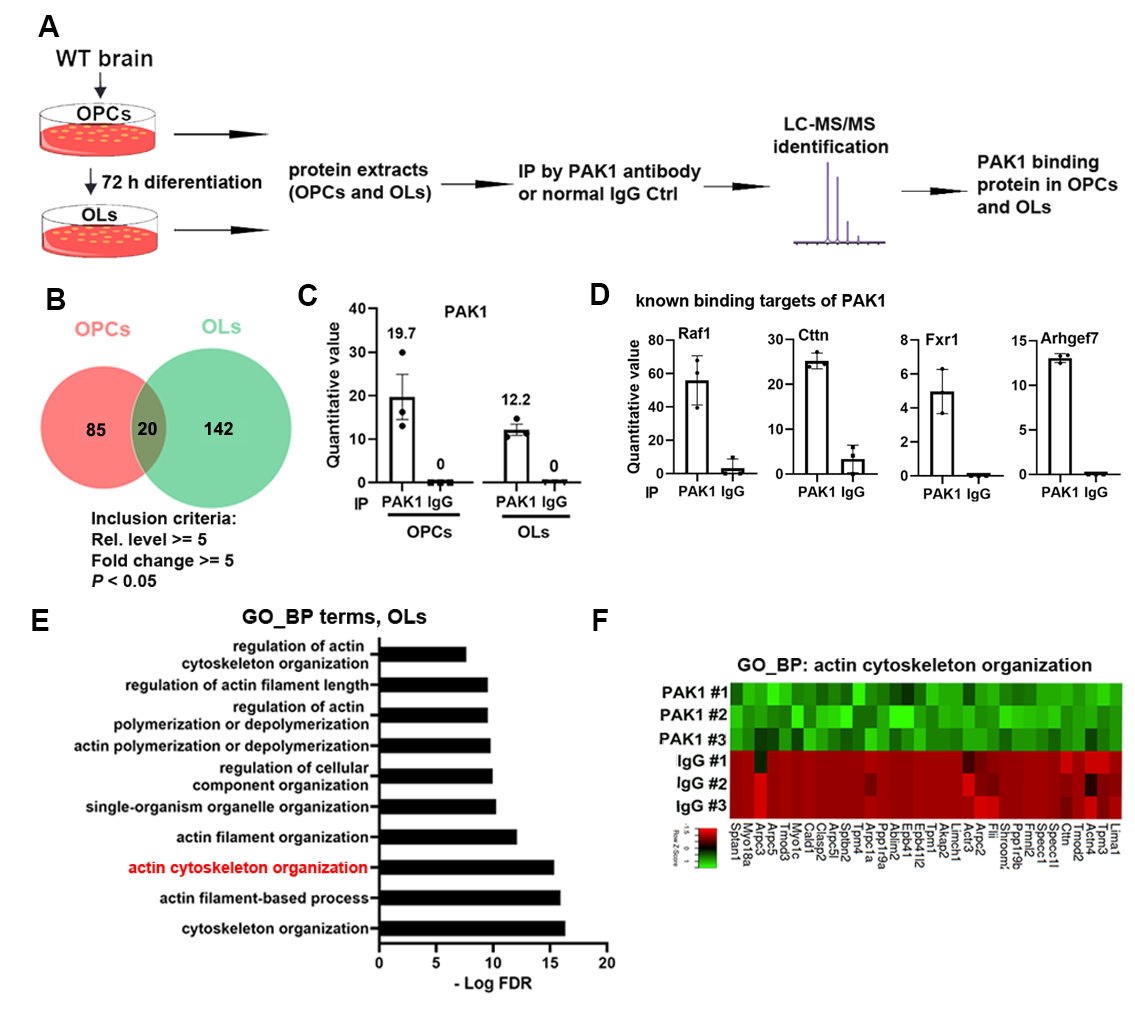

Supplement: Supplementary file 8 — Supplementary file6 (TIF 3953 KB) [file 18_2025_5728_MOESM6_ESM.tif]

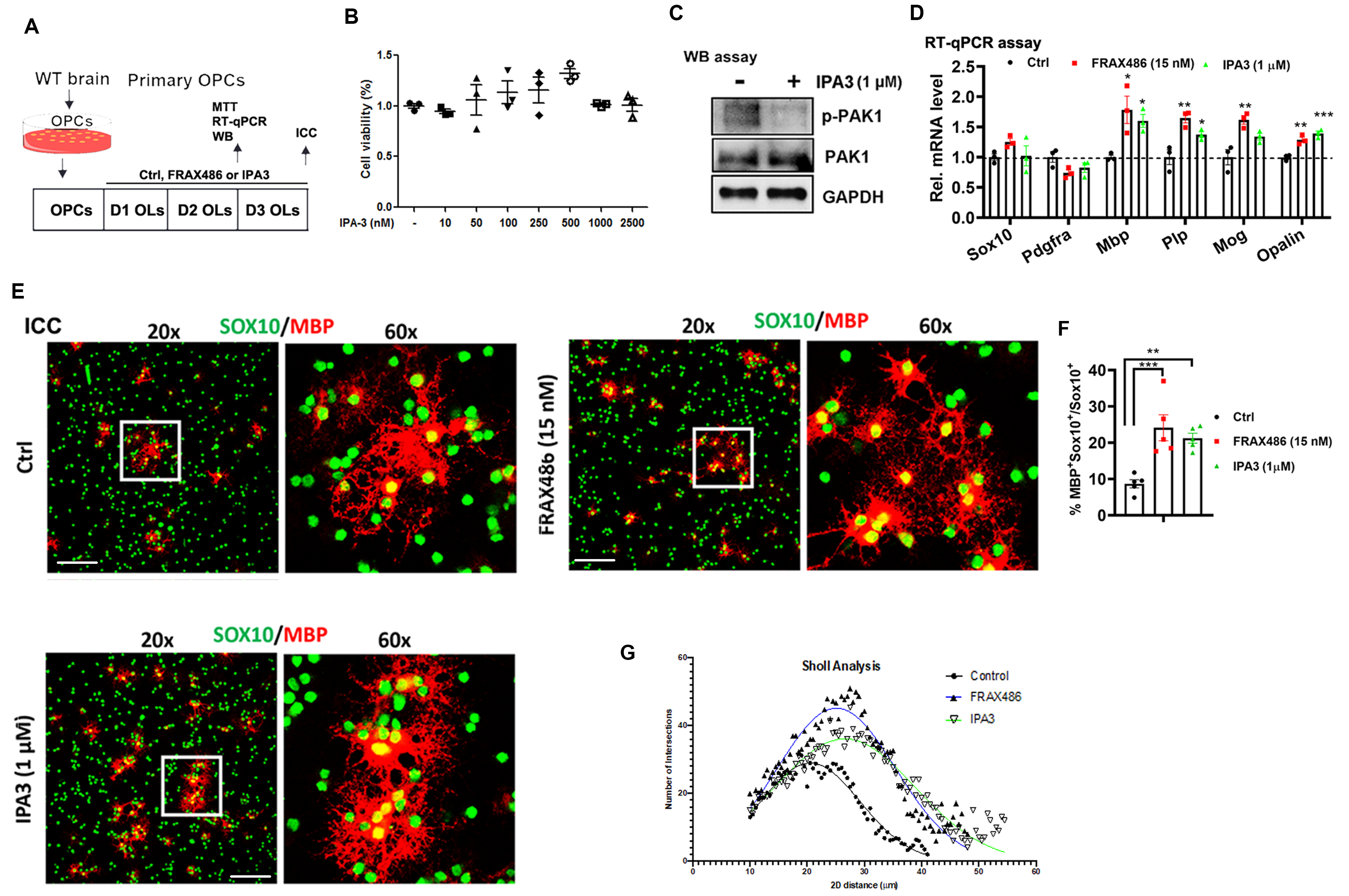

Supplement: Supplementary file 10 — Supplementary file7 (TIF 30720 KB) [file 18_2025_5728_MOESM7_ESM.tif]

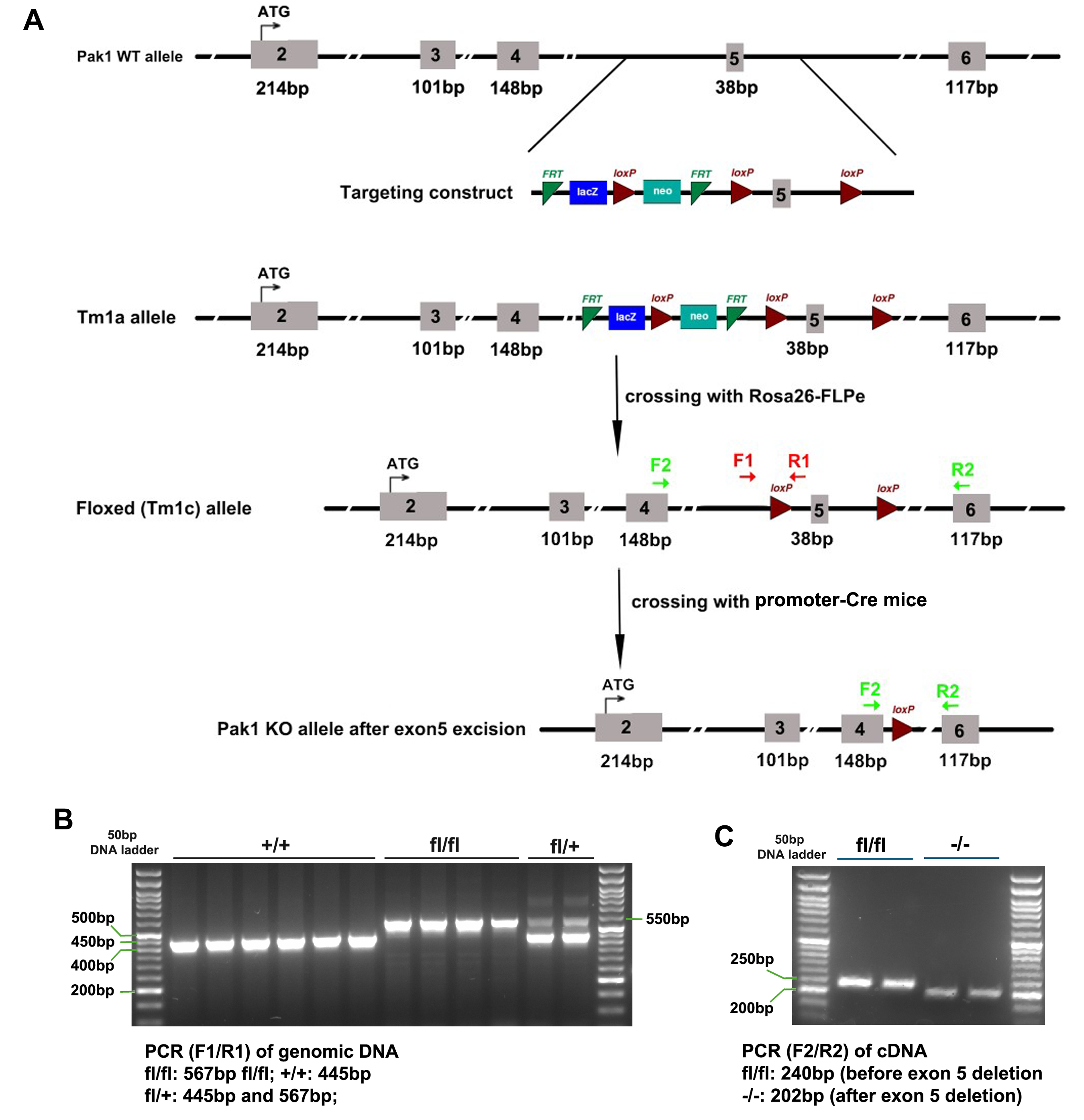

Supplement: Supplementary file 12 — Supplementary file8 (TIF 22100 KB) [file 18_2025_5728_MOESM8_ESM.tif]

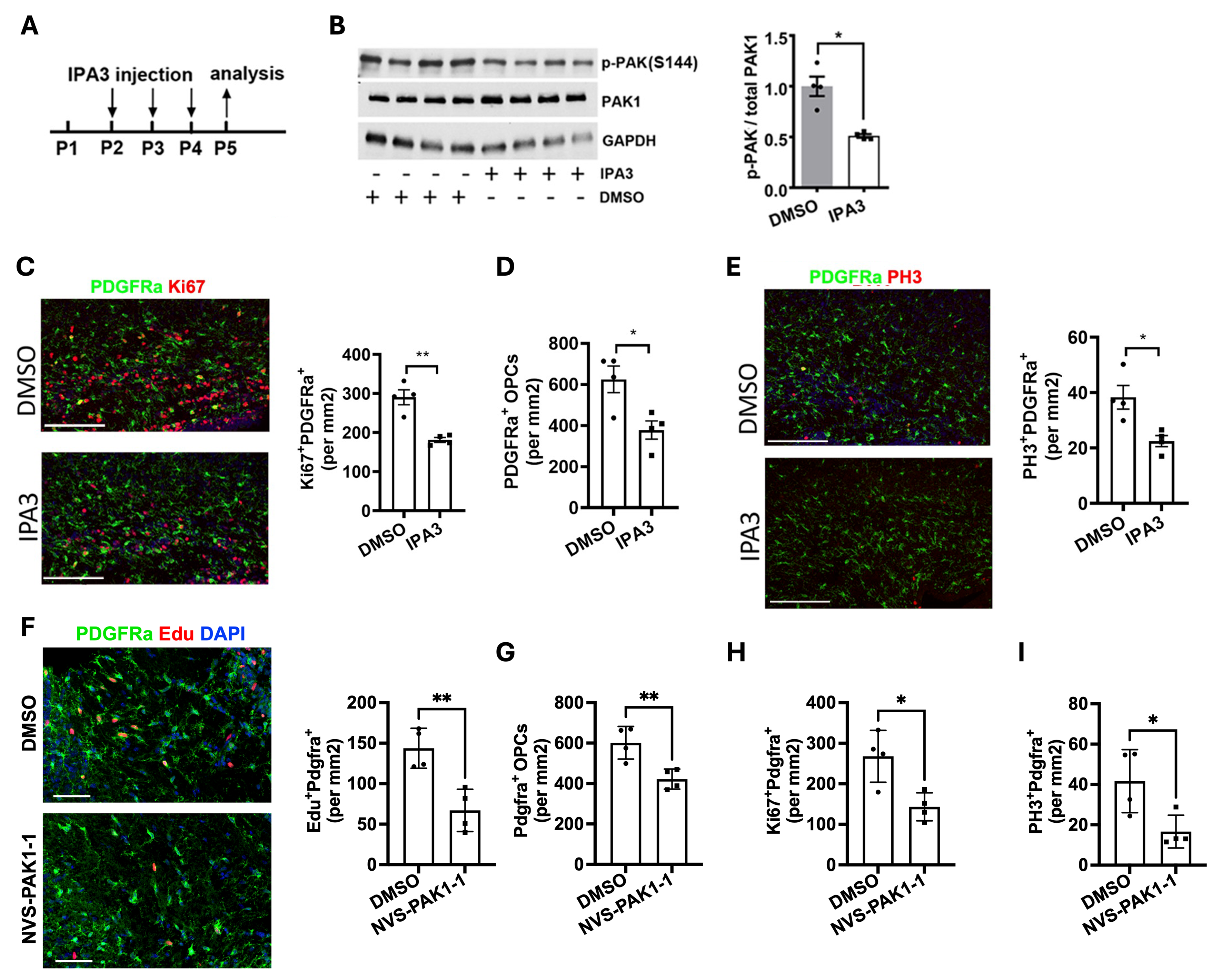

Supplement: Supplementary file 13 — Supplementary figure 5 [file 18_2025_5728_Fig9_ESM.png]

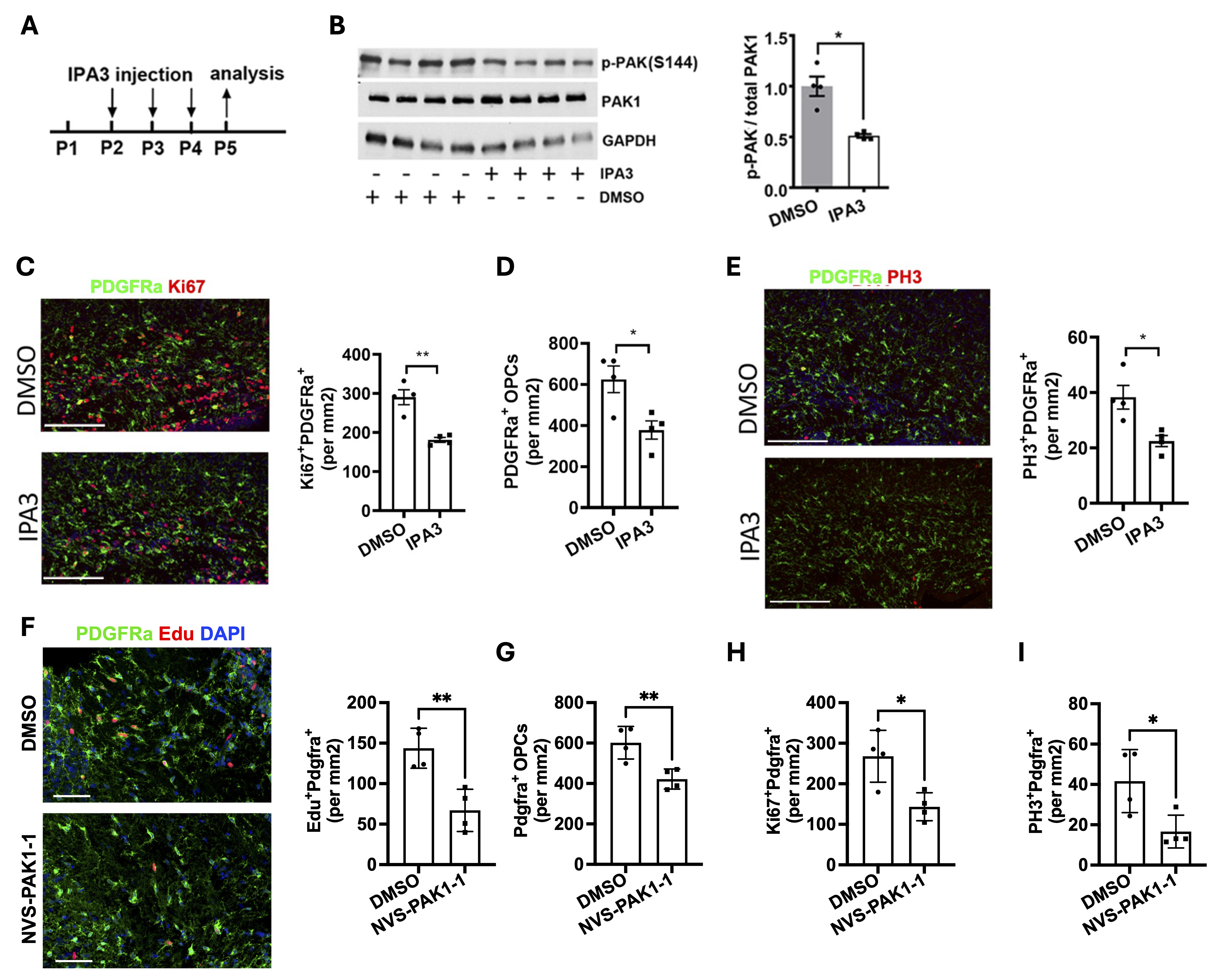

Supplement: Supplementary file 14 — Supplementary file9 (TIF 56849 KB) [file 18_2025_5728_MOESM9_ESM.tif]

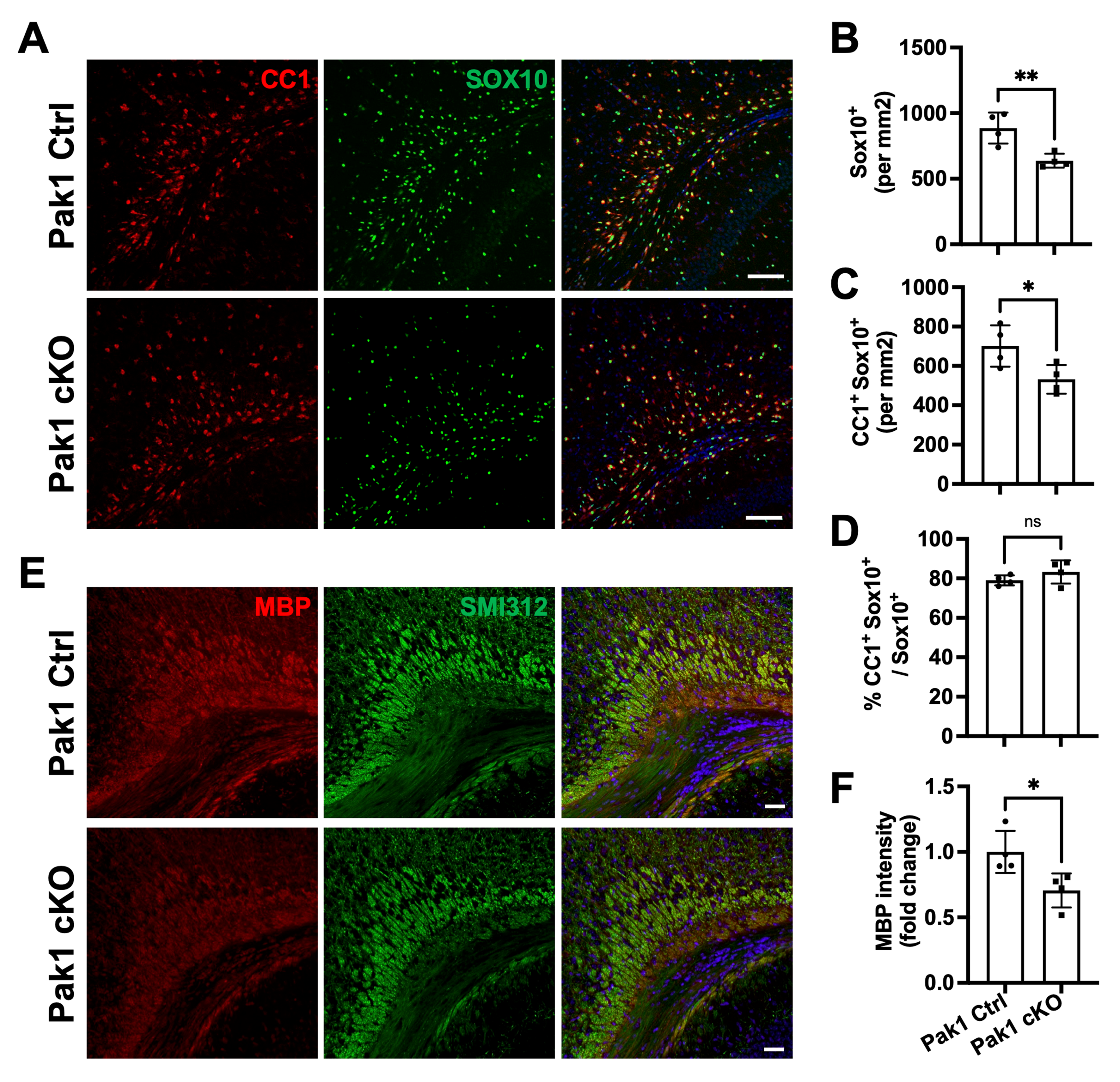

Supplement: Supplementary file 15 — Supplementary figure 6 [file 18_2025_5728_Fig10_ESM.png]

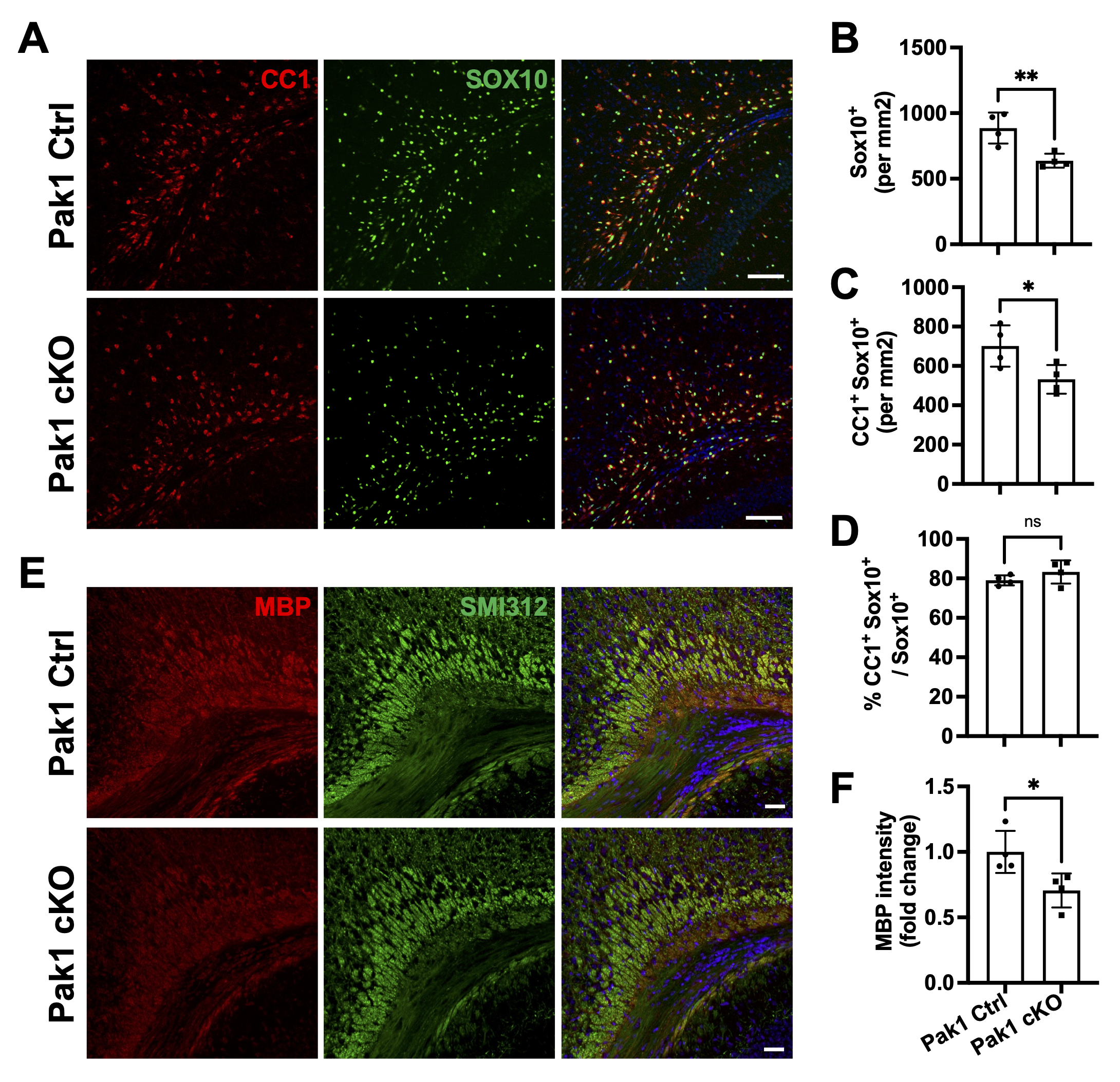

Supplement: Supplementary file 16 — Supplementary file10 (TIF 17944 KB) [file 18_2025_5728_MOESM10_ESM.tif]

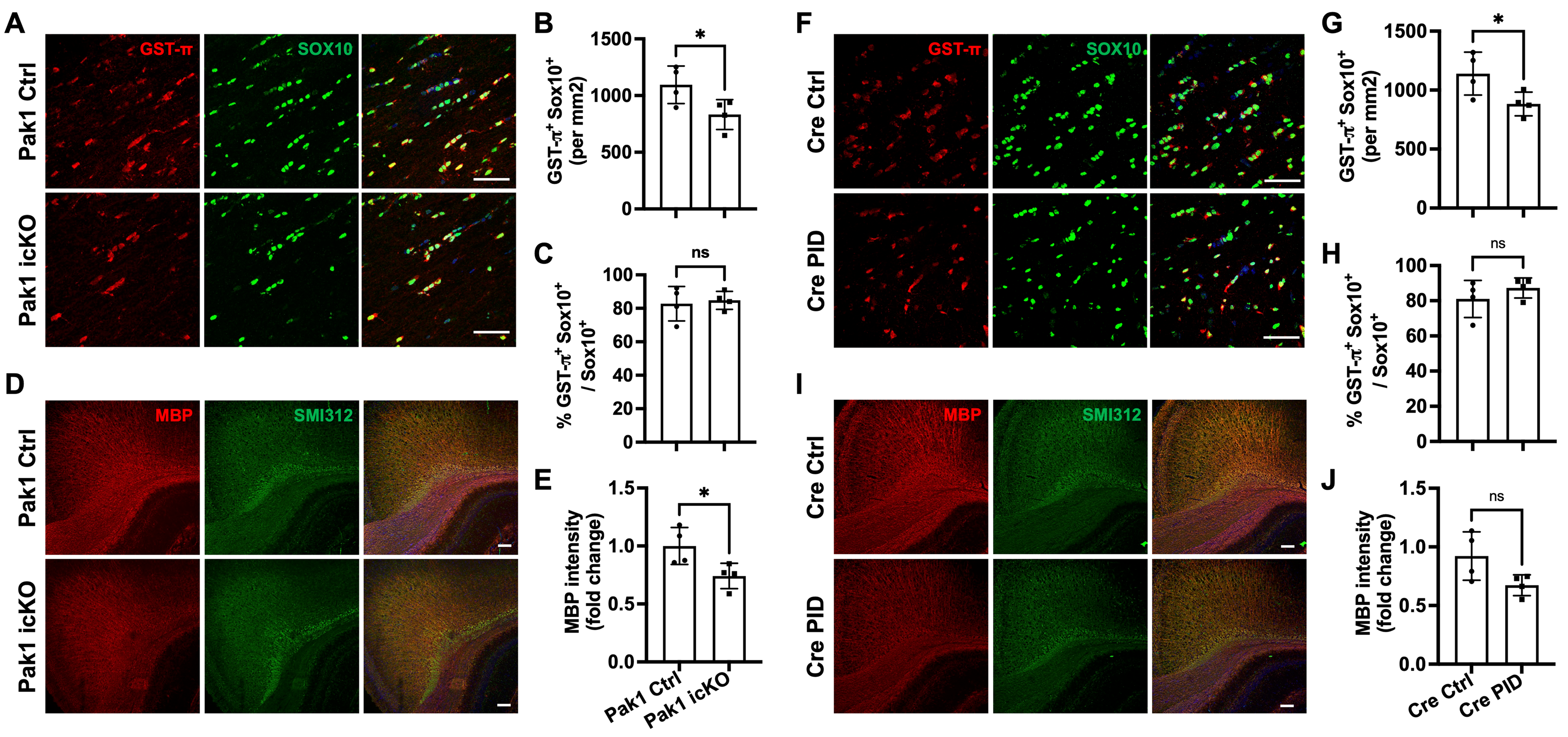

Supplement: Supplementary file 17 — Supplementary figure 7 [file 18_2025_5728_Fig11_ESM.png]

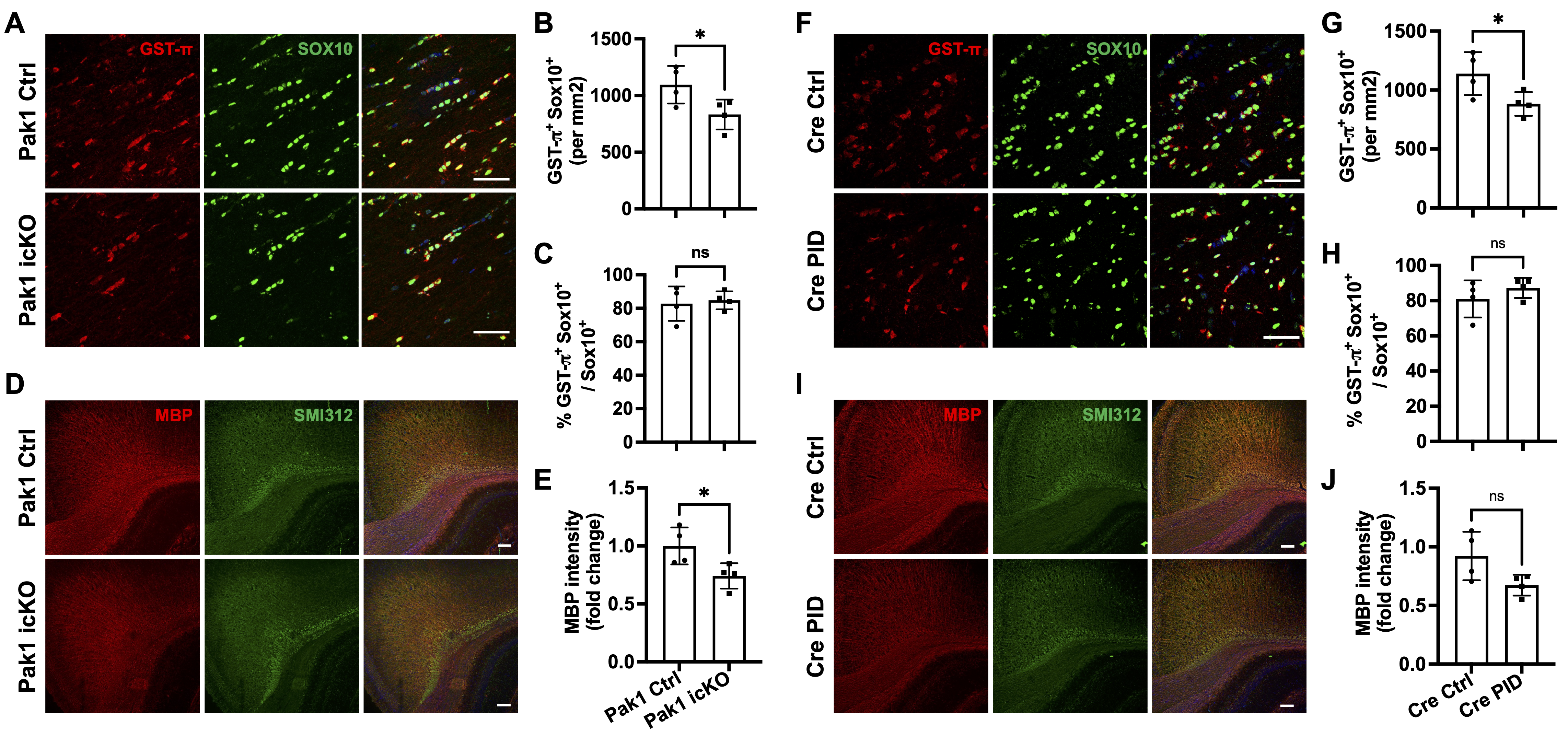

Supplement: Supplementary file 18 — Supplementary file11 (TIF 38340 KB) [file 18_2025_5728_MOESM11_ESM.tif]

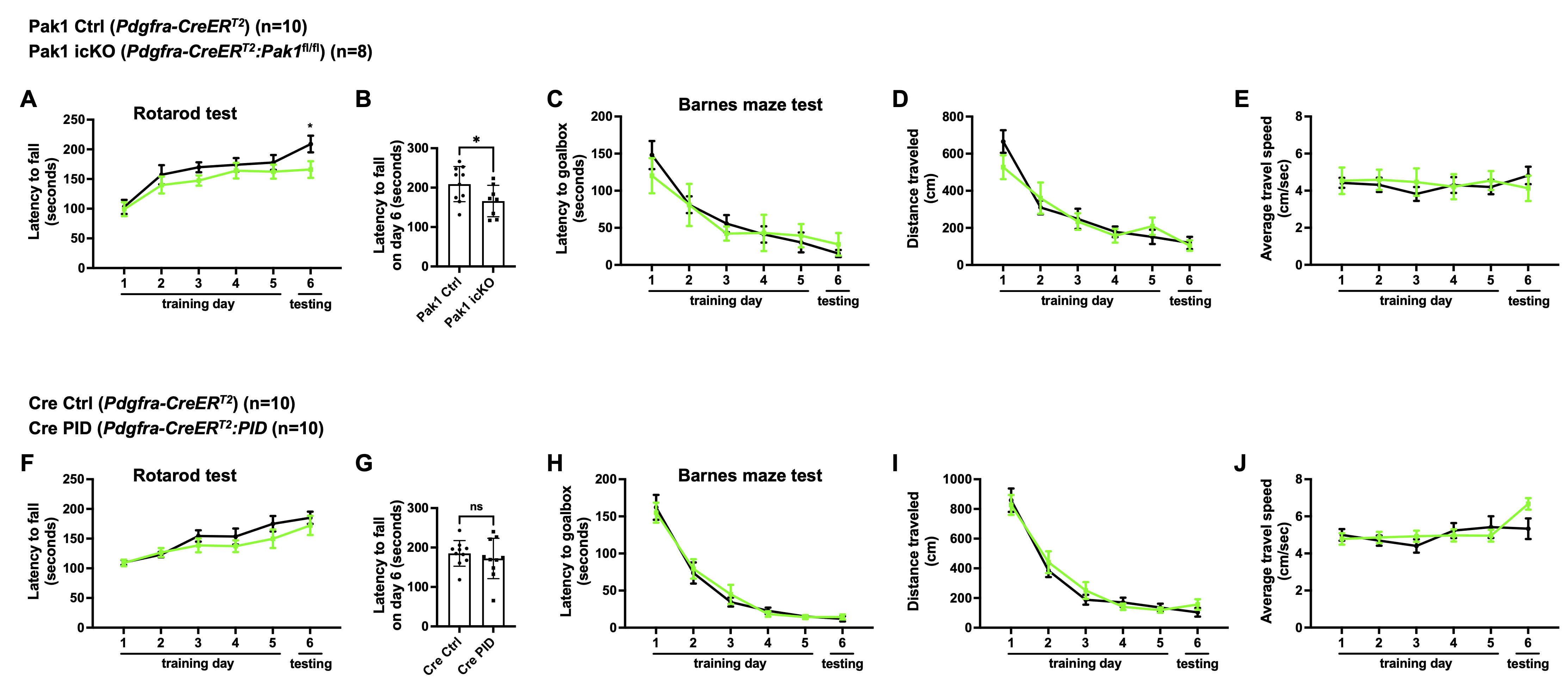

Supplement: Supplementary file 20 — Supplementary file12 (TIF 34108 KB) [file 18_2025_5728_MOESM12_ESM.tif]

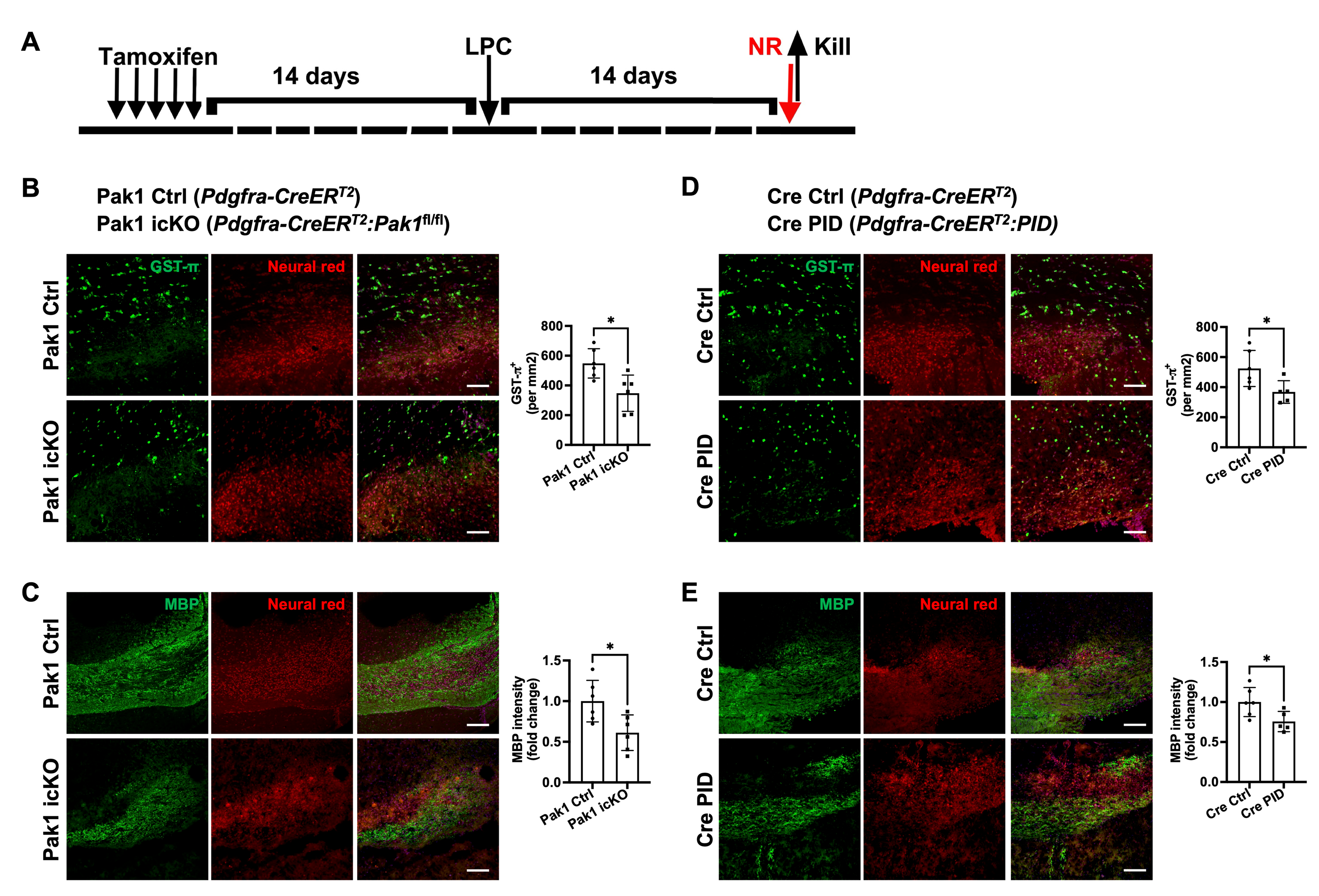

Supplement: Supplementary file 21 — Supplementary figure 9 [file 18_2025_5728_Fig13_ESM.png]

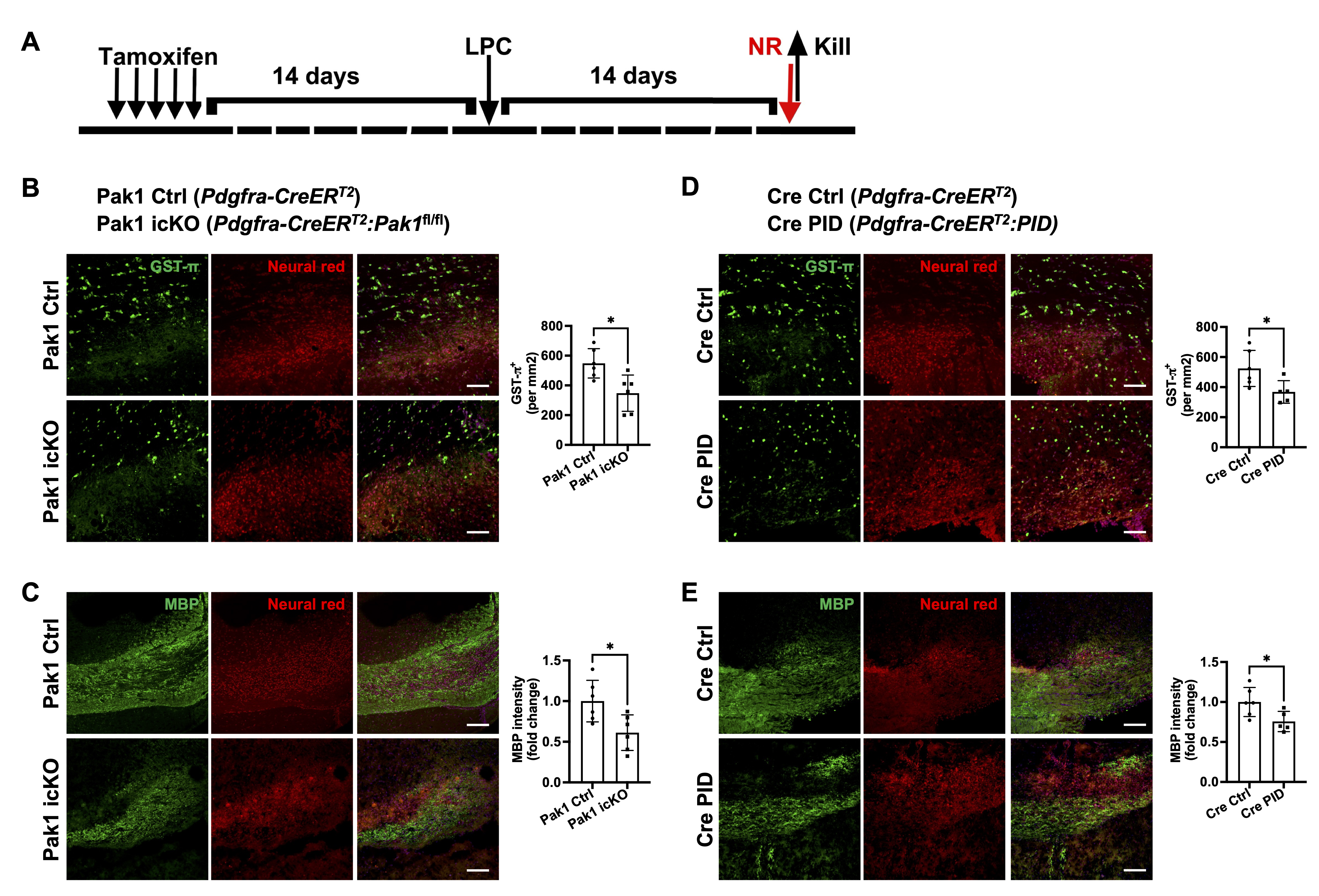

Supplement: Supplementary file 22 — Supplementary file13 (TIF 46519 KB) [file 18_2025_5728_MOESM13_ESM.tif]
